# ﻿Three new species of *Xiphocentron* Brauer, 1870 (Trichoptera, Xiphocentronidae) from Mexico

**DOI:** 10.3897/zookeys.1111.73371

**Published:** 2022-07-11

**Authors:** Joaquin Bueno-Soria, Albane Vilarino, Rafael Barba-Alvarez, Claudia Ballesteros-Barrera

**Affiliations:** 1 141 Preamble Dr., Marlton, New Jersey, 08053, USA Unaffiliated Marlton United States of America; 2 Universidade Federal de Viçosa, Museu de Entomologia, Departamento de Entomologia, Museu de Entomologia, Av. P.H. Rolfs, s/n, Campus Universitário, CEP 36570-900, Viçosa, Minas Gerais, Brazil Universidade Federal de Viçosa Minas Gerais Brazil; 3 Instituto de Biología, Departamento de Zoología, Universidad Nacional Autónoma de México, Ciudad Universitaria, 3erCto, Exterior s/n, AP 70-153, CP 04510 Ciudad de México, Mexico Universidad Nacional Autónoma de México Mexico, City Mexico; 4 Universidad Autónoma Metropolitana. Departamento de Biología, Unidad Iztapalapa, División de Ciencias Biológicas y de la Salud, AP 55–535, CP 09340 Ciudad de México, Mexico Universidad Autónoma Metropolitana Mexico, City Mexico

**Keywords:** Aquatic insects, caddisflies, Mexico, Neotropical, taxonomy

## Abstract

Three new species of the genus *Xiphocentron* (Trichoptera, Xiphocentronidae) are described from Nearctic and Neotropical regions of Mexico. Xiphocentron (Glyphocentron) flinti sp. nov. has a very unique morphology distinguished by the presence of long spines on the preapical and apical margin of tergum X. Xiphocentron (Antillotrichia) holzenthali sp. nov. is diagnosed by tergum IX, with the apical margin bearing a narrow, rounded, mesal emargination and by a spiny projection near the basal plate. These species are the first records of the family in northwestern Mexico. Xiphocentron (Antillotrichia) pineroi sp. nov. is recognized, when observed in lateral view, by its less elongate genitalia and the sinuous mesal sclerite of the inferior appendage. Additionally, we provide detailed illustrations of Xiphocentron (Antillotrichia) rhamnes Schmid, and an updated list of the distribution of the genus *Xiphocentron* in Mexico.

## ﻿Introduction

The family Xiphocentronidae Ross, 1949 is comprised of 195 species distributed in eight genera (as *Caenocentron* Schmid, 1982 was elevated to genus status) (Vilarino et al. in press). The family was erected by Ross (1949); subsequently it was placed in the family Psychomyiidae Walker, 1852 by Edwards (1961) and treated as a subfamily. Schmid (1982) in a world revision of the group resurrected the family status of Xiphocentronidae. The Xiphocentronidae are organized in two subfamilies: Proxiphocentroninae Schmid, 1982 and Xiphocentroninae Schmid, 1982 (Vilarino et al. 2018). The genus *Xiphocentron* Brauer, 1870 includes the majority of the species of the family, with 53 extant species and two subspecies widely distributed in the Neotropics (Holzenthal and Calor 2017; Vilarino and Bispo 2020) and one fossil species described from Chiapas, Mexico (Wichard et al. 2006). The genus *Xiphocentron* is subdivided into five subgenera: *Glyphocentron* Schmid, 1982, *Rhamphocentron* Schmid, 1982, *Sphagocentron* Schmid, 1982, *Xiphocentron* Schmid,1982, and *Antillotrichia* Banks, 1941 (Holzenthal and Calor 2017; Vilarino et al. 2018). The greatest diversity of subgenera is found in Mesoamerica, with only *Antillotrichia* occurring in South America and the Antilles (Vilarino and Bispo 2020). Representatives of all the subgenera occur in Mexico, and most of the distribution records of the subgenera *Rhamphocentron* and *Xiphocentron* are from Mexico. As a result of the continuing studies of the caddisfly fauna, 18 extant *Xiphocentron* and one fossil species are known to occur in Mexico (including the species here described) (Table 1). The genus *Xiphocentron* Brauer, 1870 has a wide distribution in Mexico. We have collections from the northern states of Chihuahua and San Luis Potosí, the central states of Puebla, Oaxaca, Michoacán, Ciudad de México, and Estado de México, and the southern part of the country, including the states of Veracruz, Chiapas, and Tabasco (Table 1). Because caddisflies of the genus *Xiphocentron* are diurnal (Flint 1968; Schmid 1982), often a limited number of individuals are collected when using only light traps. Rocha et al. (2017), for instance, reported that they collected two new species of *Xiphocentron*, using only the light trap method, although one of them was described with just a single type specimen.

**Table 1.** Distribution of the genus *Xiphocentron* Brauer, 1870 in Mexico. Chihuahua (Chi.), Nuevo León (NL), San Luis Potosí (SLP), Michoacán (Mich.), Estado de México (Edo. Mex.), Ciudad de México (CDMEX), Puebla (Pue.), Veracruz (Ver.), Oaxaca (Oax.), Tabasco (Tab.), Chiapas (Chis.), († fossil), (♣ New Distribution).

Species	States
X. (Xiphocentron) asilas Schmid,1982	SLP
X. (Xiphocentron) aureum Flint, 1967	Edo. Mex., ♣ Ver.
X. (Xiphocentron) bilimekii Brauer,1871	MEXICO
X. (Xiphocentron) polemon Schmid, 1982	CDMEX
X. (Xiphocentron) tarquon Schmid, 1982	Chis., Tab., Ver.
X. (Xiphocentron) chiapasi Wichard, Solórzano- Kraemer, Luer, 2006	Chis. †
X. (Xiphocentron) numanus Schmid, 1982	Oax.
X. (Sphagocentron) julus Schmid, 1982	Oax.
X. (Rhamphocentron) erato Schmid, 1982	SLP
X. (Rhamphocentron) alecto Schmid, 1982	NL, Chi., SLP
X. (Rhamphocentron) lavinia Schmid, 1982	Chis.
X. (Rhamphocentron) mexico Ross, 1949	NL, SLP, Tab.
X. (Rhamphocentron) messapus Schmid,1982	Chis.
X. (Glyphocentron) flinti sp. nov.	Chi.
X. (Antillotrichia) mezencius Schmid, 1982	Pue.
X. (Antillotrichia) rhamnes Schmid, 1982	Mich., Oax.
X. (Antillotrichia) serestus Schmid, 1982	Mich., Oax.
X. (Antillotrichia) holzenthali sp. nov.	Chi.
X. (Antillotrichia) pineroi sp. nov.	Tab.

## ﻿Methods

The specimens of the genus *Xiphocentron* studied here were borrowed from the collections of the National Museum of Natural History, Smithsonian Institution in Washington, DC, and from the Colección Nacional de Insectos, Instituto de Biología de la Universidad Nacional Autónoma de México.

For the description of wing venation, we followed Vilarino and Bispo (2020). For the study of the internal structure of the male genitalia, we put the entire adults or an abdomen into a small container with a solution of 10% of KOH, and then kept on a hot plate at 100 °C for 10 minutes, in order to clear the genitalia. After that, the specimens were kept in 10% acetic acid for 10 min to stop the clearing reaction (Prather 2003). Subsequently, the specimens were placed on microscope slides with a drop of glycerin for the observation of the male genitalia. We used a dissection microscope (LEICA Model EZ4) and a ZEISS compound microscope with camera lucida for observation and creation of the drawings, the latter subsequently digitized on the computer using Adobe Illustrator CS6. Morphological terminology and style of the description of the male genitalia, follows that presented by Muñoz and Holzenthal (1997) and Schmid (1982). Distribution maps were generated using ArcGIS v. 10.2 (ESRI 2013). Distributional data for Xiphocentronidae was compiled from the literature.

The type materials are deposited as indicated in each species description, in the collections: National Museum of Natural History, Smithsonian Institution in Washington, DC (USNM), and Colección Nacional de Insectos, Instituto de Biología de la Universidad Nacional Autónoma de México (CNIN, formerly IBUNAM).

## ﻿Results

### ﻿Family Xiphocentronidae Ross, 1949

**Genus *Xiphocentron* Brauer, 1870**

#### ﻿ Xiphocentron (Glyphocentron) flinti

Taxon classificationAnimaliaTrichopteraXiphocentronidae

Bueno, Vilarino & Barba sp. nov.

BB2E459B-37A0-5BFC-A5B0-DA8373144C69

http://zoobank.org/5BE15179-7CA0-4F3D-A475-D275022D2985

Figures 1 , 2 , 6

##### Diagnosis.

This new species is very distinct from all other *Xiphocentron* species. The group of long, mesally situated setae on the basal portion of the inferior appendages has some resemblance to species in the subgenus Xiphocentron, whereas the complex tergum X is similar to species in the subgenus Glyphocentron. Xiphocentron (G.) flinti sp. nov. can be distinguished from all the species of the family by the unique tergum X which bears long spines on the preapical and apical margin and visible in both dorsal and ventral views.

##### Description.

**Figure 1.**
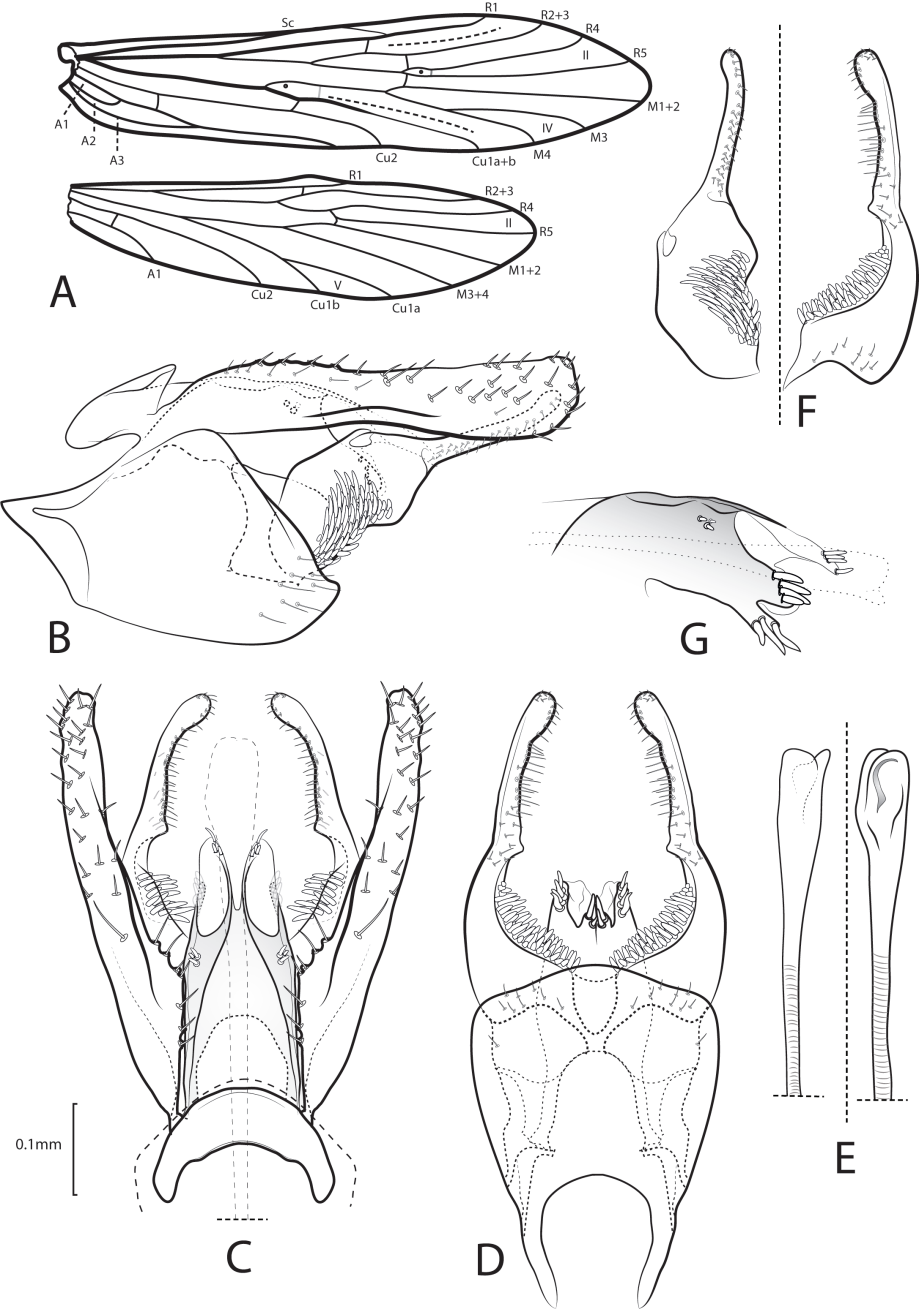
Xiphocentron (Glyphocentron) flinti sp. nov., holotype, adult, ♂ **A** forewing and hindwing. Male genitalia **B** left lateral **C** dorsal **D** ventral **E** phallus, apex in lateral and dorsal view **F** detail of right inferior appendage mesal surface, lateral and ventral view **G** detail of segment X left lateral.

**Adult.** Forewing length 4.8–9.0 mm, *n* = 16; fork II and fork IV present; Sc reaching C subapically, meeting R1 apically; fork II sessile at discoidal cell, with crossvein between R5 and M1+2; thyridial cell shorter than discoidal cell; three anal veins present (Fig. 1). Hindwing with fork II and fork V present. Color fuscous. Tibial spur formula 2–4-3. Hind tibia apical spurs not modified. Sternum V bearing pair of reticulated regions.

***Male genitalia*.** Tergum IX small, ellipsoidal; in lateral view, rounded ventrally, narrow dorsally, anterior margin straight; in dorsal view anterior margin with wide, deep, U-shaped mesal emargination; apical margin rounded. Sternum IX subtriangular, in lateral view, about two times as long as high; anterior margin with elongate, wide, mesal apodeme; ventral margin rounded; posterior margin rounded; in ventral view, anterior margin with deep, U-shaped emargination, enlarging apically; posterior margin rounded. Tergum X in lateral view, subrectangular, narrow basally, wide mesally, with a group of spinelike setae, preapically rectilinear, apically bifurcated in two lobes, ventral lobe longer with group of long spinelike setae, dorsal lobe shorter with apical spinelike setae, anterior margin curved; in dorsal view, subtriangular, wide basally, narrow apically, lateral surface sclerotized, mesally membranous; apex with deep cleft, forming two rounded membranous lobes, bearing preapical spinelike setae, apices divergent; in ventral view, apex with two long mesal spinelike setae. Preanal appendages long, dorsal margin crenulate, in lateral view, broad, parallel-sided, with longitudinal ridge, apex rounded; in dorsal view, enlarged basally, narrowed at middle. Inferior appendage short, approximately half length of preanal appendages, basal section with a group of long spinelike setae; in lateral view, basal section broad, apical section narrow; in ventral view, basal section subtriangular, with a line of large spinelike setae, apical section long and thin, curved mesad; basal plate short, shorter than half sternum IX length. Phallus, long, slender, tubular, apex enlarged, with narrow curved sclerite.

**Figure 2.**
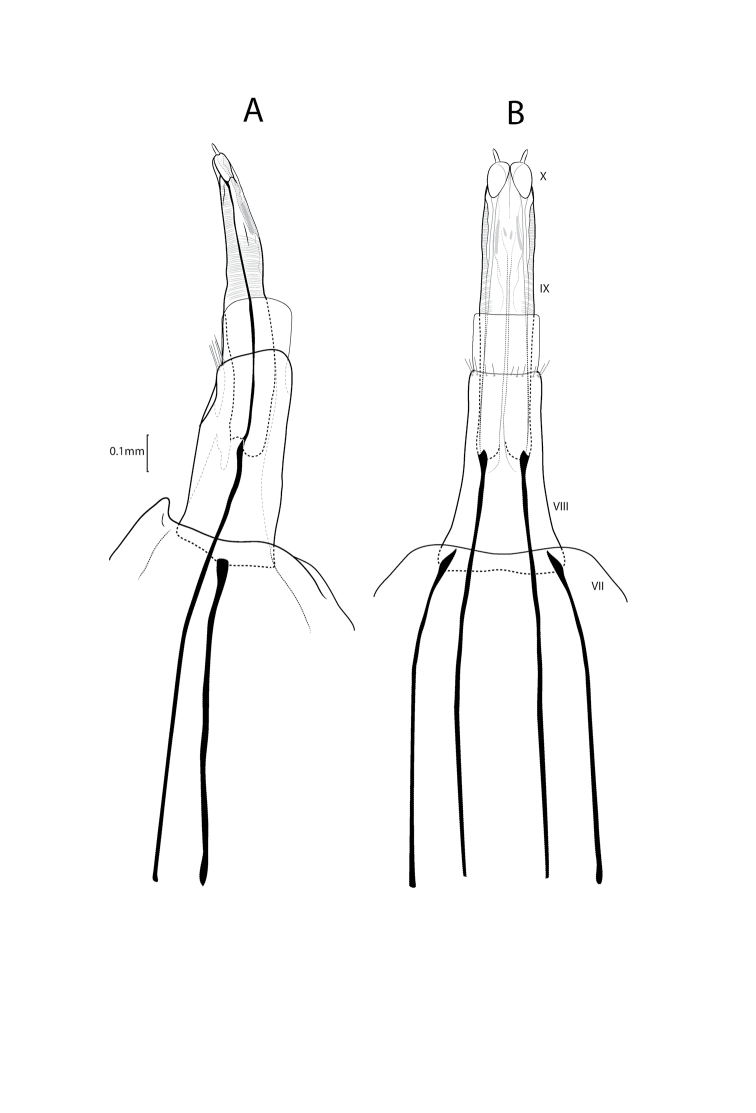
Xiphocentron (Glyphocentron) flinti sp. nov., paratype, adult, ♀, genitalia **A** lateral **B** dorsal view.

***Female genitalia*.** VIII segment long and narrow, synscleritous dorsally, internally with a pair of long slender apodemes from anterior margin; intersegmental membrane nearly as long as IX segment when extended. IX segment long and slender, with a pair of internal apodemes arising from anterior margin; apex with a pair of slender processes.

##### Type material.

***Holotype***: ♂ **Mexico, Chihuahua**, Ruta San Rafael-Cuiteco, 27°26'13"N, 108°00'32"W, elev. 1707 m, 30.VIII.2005, J. Bueno and R. Barba leg. pinned with abdomen in glycerin (CNIN). ***Paratypes***: ibid., Chihuahua, Riito, Hwy. 16, 10 mi E. Yepachic 28°10'26"N, 108°10'36"W, elev. 2086 m, 28.VI.1987, R. Baumann, B. Kondratieff, Sargent and Wells leg. 8♂ 8♀ in alcohol (USNM); ibid., small stream Cascada de Basaseachic, 28°10'52"N, 108°12'44"W, elev. 1950 m, 28.VI.1987, B. Kondratieff and R. Baumann leg. 1♂ in alcohol (USNM); ibid., Cascada de Basaseachic, 22.VIII.1986, B. Kondratieff leg. 1♂ pinned (USNMENTO1518156); ibid., fork Arroyo Bandera near Jct. Río Chuhuichupa, 25.VI.1987, B. Kondratieff and R. Baumann leg. 1♂ 1♀ in alcohol (USNM).

##### Etymology.

We dedicated this species, with sadness and love, to the memory of a great entomologist, Dr Oliver S. Flint Jr, who passed away on May 18, 2019.

##### Distribution.

All the specimens were collected at Sierra Tarahumara, the mountain region of Chihuahua State (Fig. 6).

##### Remarks.

The affinities of Xiphocentron (Glyphocentron) flinti sp. nov. are not very clear. The preanal appendages present a mesal ridge, a character present in the species of the genus *Melanotrichia*. The long setae on the basal portion of the inferior appendages in this species are similar to species within the subgenus X. (Xiphocentron) or even to Cnodocentron (Caenocentron). The complex tergum X with apical points puts it closer to the subgenus X. (Glyphocentron). The new species lacks other diagnostic characters of *Melanotrichia* (fan-like spine line), *Cnodocentron* (bifurcate inferior appendage), or X. (Xiphocentron) (modified hind leg spurs, and presence of forewing fork I); therefore, we are placing it within subgenus X. (Glyphocentron), for which the diagnostic character is the presence of points on tergum X (Schmid 1982).

#### ﻿ Xiphocentron (Antillotrichia) holzenthali

Taxon classificationAnimaliaTrichopteraXiphocentronidae

Bueno, Vilarino & Barba sp. nov.

D7547D53-EED2-5E38-801D-90C7F39AFE31

http://zoobank.org/919102E4-F494-4B38-8FD2-C327ECC42FCE

Figures 3 , 6

##### Diagnosis.

This new species is very similar to Xiphocentron (Antillotrichia) serestus Schmid, 1982. However, Xiphocentron (Antillotrichia) holzenthali sp. nov. can be separated from X. (Antillotrichia) serestus by the shape of the tergum IX, as viewed dorsally; in X. (Antillotrichia) holzenthali sp. nov. the apical margin has a narrow, rounded, mesal emargination, while in X. (Antillotrichia) serestus the mesal emargination is wide and shallow. In the new species, the apical margin of sternum IX, in ventral view, has a narrow, rounded, mesal emargination, while in X. (Antillotrichia) serestus this margin has a trilobed mesal emargination. Also, in the new species the inferior appendage, in ventral view, has a spiny projection near the basal plate, which is absent in X. (Antillotrichia) serestus.

**Figure 3.**
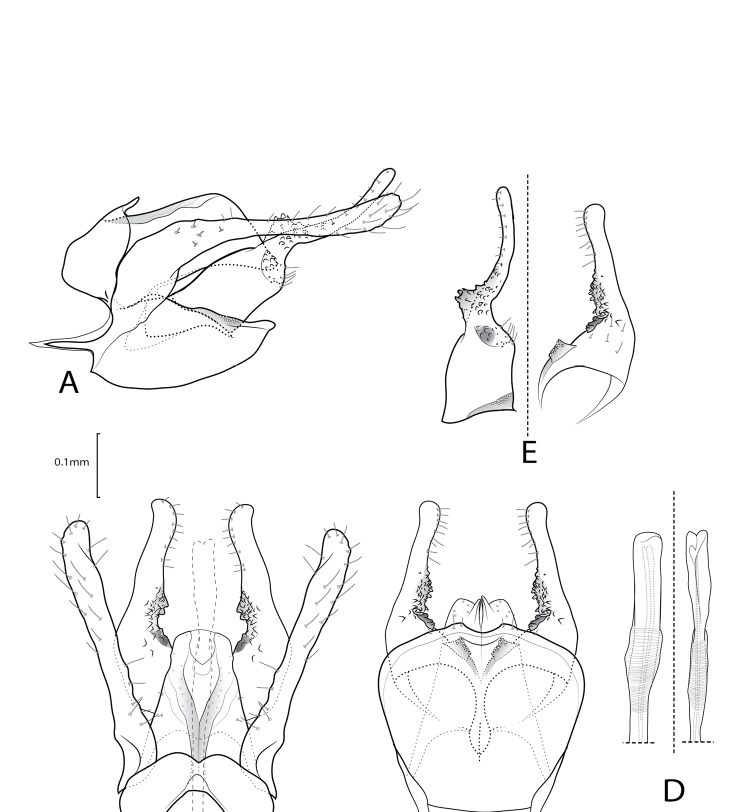
Xiphocentron (Antillotrichia) holzenthali sp. nov., holotype, adult, ♂, genitalia **A** left lateral **B** dorsal **C** ventral **D** phallus left lateral and dorsal **E** detail of right inferior appendage mesal surface, lateral and ventral view.

##### Description.

**Adult.** Forewing length 6–7 mm, *n* = 5. Color in alcohol pale. Tibial spur formula 2–4-3. Hind tibia apical spurs not modified. Sternum V bearing pair of reticulated regions.

***Male genitalia*.** Tergum IX semicircular; in lateral view, wide basally, narrow apically; dorsal margin curved; ventral margin nearly straight; in dorsal view anterior margin with deep V-shaped central incision; apical margin with narrow, rounded, mesal emargination. Sternum IX ovate, in lateral view, about twice as long as high; anterior margin with slender, pointed, mesal apodeme; ventral margin rounded; dorsal margin subtriangular; in ventral view, enlarging preapically; anterior margin rectilinear; posterior margin with small mesal emargination. Tergum X in lateral view, rectangular, narrow, acute apically; in dorsal view, subtriangular, wide basally, narrow apically; lateral surface sclerotized, each sclerotized side fused mesally; apex with deep, V-shaped emargination; in ventral view, subtriangular, wide basally, narrow, cleft apically. Preanal appendages long, margins crenulate, surface weakly setose; in lateral view, broad basally, parallel-sided, narrow preapically, apex rounded. Inferior appendages, in lateral view long, approximately same length as preanal appendages, weakly setose; widest basally and mesally, apical section narrower, slender, upturned, and curved; mesal surface, with short, thick, peglike setae, separated in two, small patches, visible in ventral and dorsal view; in ventral view with spiny projection near basal plate; basal plate long, about as long as half sternum IX length. Phallus long, slender, tubular, slender apically.

***Female genitalia*** (not illustrated). VIII segment narrowly divided dorsally, internally with pair of long slender apodemes from anterior margin; intersegmental membrane nearly as long as IX segment when extended. IX segment long and slender with pair of internal apodemes arising from anterior margin; apex with pair of slender processes.

##### Type material.

***Holotype***: ♂ **Mexico: Chihuahua**, Jct. E & W Forks Arroyo Toro, Toro Basin 28°06'35"N, 107°37'28"W, elev. 2425 m, 23.VI. 1987, B. Kondratieff and R. Baumann leg. in glycerin (USNM). ***Paratype*s**: ibid., 1♂1♀ in glycerin (USNM); Chihuahua, Arroyo Chuchupate, Trib. Río Chuhuichup 28°48'08"N, 107°24'43"W, elev. 2426 m, 23.VI.1987, B. Kondratieff and R. Baumann leg. 2♂ in glycerin (USNM).

##### Etymology.

We name this species in honor of Dr Ralph Holzenthal in recognition of his great contribution to the knowledge of the systematics and distribution of Neotropical caddisflies.

##### Distribution.

All the specimens were collected at Sierra Tarahumara, the mountainous region of Chihuahua State (Fig. 6).

#### ﻿ Xiphocentron (Antillotrichia) pineroi

Taxon classificationAnimaliaTrichopteraXiphocentronidae

Bueno, Vilarino & Barba sp. nov.

13B7AC63-C68C-5FAD-9D2D-EEC63ABCA5B1

http://zoobank.org/27C736E6-3A0A-4383-A5CF-A15EB24D1612

Figures 4 , 6

##### Diagnosis.

This new species is similar to other species with a mesal sclerite on the inferior appendages. The new species is particularly similar to Xiphocentron (Antillotrichia) surinamense Flint, 1974, and Xiphocentron (Antillotrichia) pintada Flint, 1983 due to the shape of tergum IX and the mesal sclerite. Xiphocentron (Antillotrichia) pineroi sp. nov. can be distinguished from X. (Antillotrichia) surinamense by the longer and thinner sternum IX, preanal and inferior appendages in lateral view. It is distinguished from X. (Antillotrichia) pintada by its longer, sinuous mesal sclerite in lateral view, and by the deeper mesal emargination of sternum IX in dorsal view.

**Figure 4.**
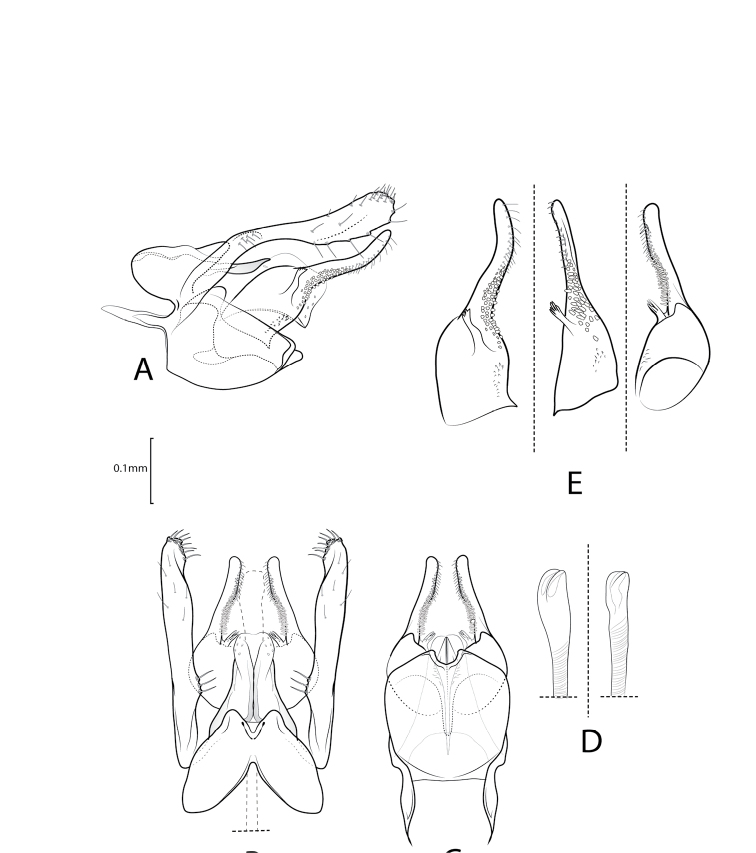
Xiphocentron (Antillotrichia) pineroi sp. nov., holotype, adult, ♂, genitalia **A** left lateral **B** dorsal **C** ventral **D** phallus apex, left lateral and dorsal **E** detail of right inferior appendage mesal surface, lateral, ventro-lateral, and ventral view.

##### Description.

**Adult.** Forewing length 6.0 mm. Color in alcohol pale. Tibial spur formula 2–4-3. Hind tibia apical spurs not modified. Sternum V bearing pair of reticulated regions.

***Male genitalia*.** Tergum IX in lateral view, ovate, anterior margin rounded, posterior margin rectilinear, dorsal margin produced posterad. In dorsal view anterior margin with narrow, V-shaped, mesal emargination. Apical margin with acute mesal emargination. Sternum IX ovate, in lateral view, about twice as long as high; anterior margin with elongate, slender, pointed, mesal apodeme; ventral margin convex; dorsal margin subtriangular; in ventral view, anterior margin rectilinear; posterior margin with short, rounded, mesal emargination. Tergum X, in lateral view, cylindrical, narrow basally, mesally wider, circular apically. In dorsal view, subtriangular, wide basally, narrow apically; lateral surface sclerotized, each sclerotized side fused mesally; apex with deep, V-shaped emargination, forming two lobes; in ventral view, subtriangular, wide basally, narrow and cleft apically, forming two apicomesal projections. Preanal appendages elongated, about twice as long as segment X and setose. In lateral view, basally directed posterodorsally, then bent posterad, constricted at mid-length, apex rounded. In dorsal view, narrowed at base and sinuous, rectangular preapically, apex rounded, rugose. Inferior appendages long, shorter than preanal appendages, basal section with narrow and sinuous sclerite bearing small spines at apex; in lateral view, basal section broad, apical section longer than basal region, slender; in dorsal view, apex rectangular, basal section rounded; mesal surface with row of several short spine-like setae and narrow sclerite; in ventral basal section rugose near basal plate; basal plate long, about as long as half sternum IX length. Phallus long, slender, tubular, apex enlarged.

**Female.** Unknown.

##### Type material.

***Holotype***: ♂ **Mexico: Tabasco**, Mpio. Huimanguillo Ejido Villa de Guadalupe 1a Secc. Cascada Cerro de Las Flores Rta. Malpasito-Carlos A. Madrazo 17°21'39"N, 93°37'29"W, elev. 540 m, 16.III.2000, J. Bueno, R. Barba, A. Rojas leg. in glycerin (CNIN).

##### Etymology.

We take great pleasure in naming this species for Dr Daniel Ignacio Piñero-Dalmau in recognition of his great contributions to the knowledge of the genetics of populations and conservation of Mexican plants.

##### Distribution.

The holotype was collected at a waterfall in a rain forest in Tabasco State (Fig. 6).

#### ﻿ Xiphocentron (Antillotrichia) rhamnes

Taxon classificationAnimaliaTrichopteraXiphocentronidae

Schmid, 1982

171E2D5B-DECF-5CA7-AD7A-5B451BCB9818

Figures 5 , 6

##### Material analyzed.

**Mexico: Veracruz**, N. Huatusco, 19°8'53"N, 96°58'1"W, elev. 1344 m, 31.VII.1966, O.S. Flint and M.A. Ortiz leg. 1♂ pinned (USNMENTO1028628) [holotype]. **Estado de México**, Mpio. Villa de Allende, km 60 Carr. Toluca-Valle de Bravo San Cayetano, 19°22'14"N, 100°5'15"W, elev. 2516 m, 13.VI.2003, M. Razo and L. Oñate leg. 2♂ pinned (CNIN). **Puebla**, Mpio. Progreso, Río San Juan 5.8 km N de Tlatlauquitepec, 19°50'14"N, 97°30'48"W, elev. 2003 m, 28.VI.1996, A. Contreras and R. Barba leg. 1♂ in glycerin (CNIN). **Oaxaca**, Santa María de Yavesia, 17°13'36"N, 96°25'35"W, elev. 2062 m, 16.VIII.2001, J. Bueno, R. Barba and A. Ibarra leg. 9 ♂♂ in glycerin (CNIN) [**specimen illustrated**]. **Veracruz**, Altotonga, Río Pancho Pozas 19°44'42"N, 97°14'52"W, elev. 2008 m, 25.VII.1994, B. Kondratieff and R. Baumann leg. 1♂ in glycerin (CNIN).

**Figure 5.**
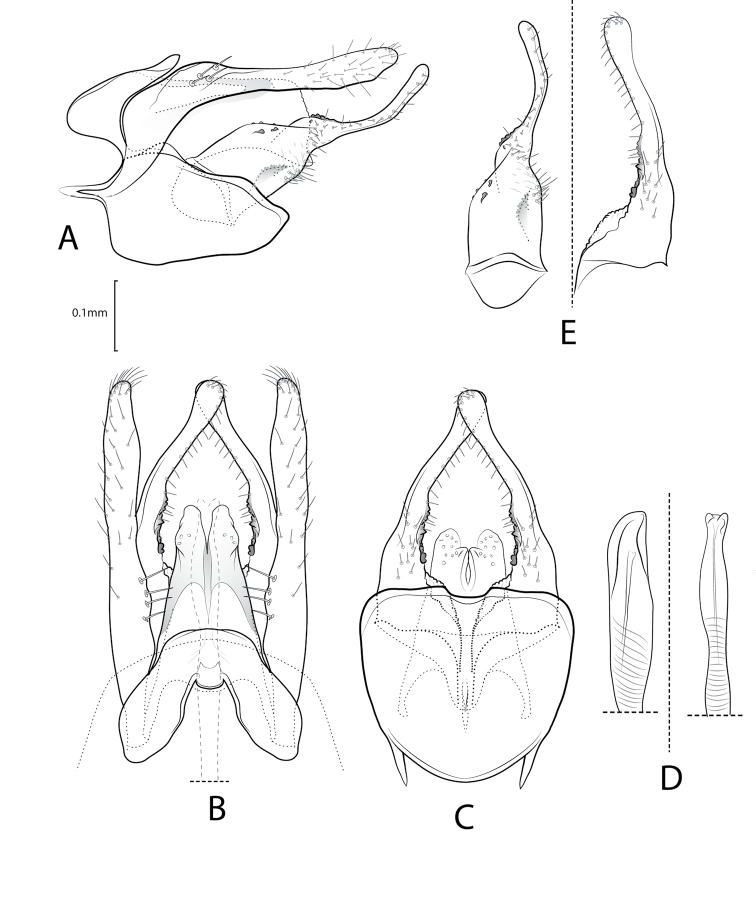
Xiphocentron (Antillotrichia) rhamnes Schmid, 1982, adult, ♂, genitalia **A** left lateral **B** dorsal **C** ventral **D** phallus apex, left lateral and dorsal **E** detail of right inferior appendage mesal surface, lateral, and ventral view.

##### Distribution.

**Figure 6.**
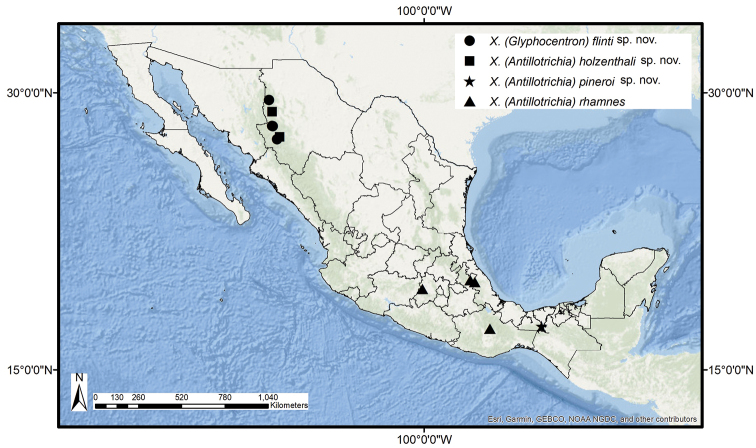
Mexican distribution of the new species of *Xiphocentron* and X. (Antillotrichia) rhamnes Schmid, 1982.

Mexico; Estado de México, Puebla, Oaxaca, and Veracruz states (Fig. 6).

##### Remarks.

In the original description, Schmid (1982) did not provide the full depiction of the dorsal and ventral view of the male genitalia of this species. Therefore, some characters cannot be observed, such as the shape of the anterior margin of the tergum IX in dorsal view and the basal crenulate area of the inferior appendage in ventral view. The holotype is fixed in a permanent slide in dorso-lateral view. To avoid damaging it, we illustrated another identified specimen (from Oaxaca) and analyzed the holotype with material from the same province of the holotype (Veracruz) and other regions of Mexico.

## ﻿Discussion

According to the biogeographic provinces proposed by Morrone et al. (2017), X. (Antillotrichia) holzenthali sp. nov. and X. (Glyphocentron) flinti sp. nov. are distributed in the Nearctic region of Mexico (Fig. 6), particularly in the province of Sierra Madre Occidental (in the Gran Meseta and Cañones Chihuahuenses and Sierras and Subcañadas del Norte subprovinces). This province presents the largest mountain system in the country, with altitudes of 2000–2500 m a.s.l. (Morrone et al. 2017). The collection sites of X. (Antillotrichia) holzenthali sp. nov. are located at 2060 m on average, and X. (Glyphocentron) flinti sp. nov. at 1809 m. Both species are distributed in places with Subhumid Temperate Climate (Cw) (García and CONABIO 1998) and vegetation consisting of conifer and oak forests. These species are the first representatives of the family Xiphocentronidae recorded from northwestern Mexico. The biogeographical analysis of *Caenocentron* suggests that these western mountain ranges were an important dispersal area of early radiations during the Oligocene (Vilarino et al. in press); this might also be true for the radiation of other groups within Xiphocentronidae. The distribution of X. (Antillotrichia) rhamnes is found within the Mexican Transition Zone in the Transverse Volcanic Province and the Province of Sierra Madre del Sur, at an average altitude of 2133 m, which is characterized by a subhumid temperate climate (Cw) and vegetation commonly consisting of coniferous and oak forests. Xiphocentron (Antillotrichia) pineroi sp. nov. is the southernmost occurring of these species and is distributed in the Neotropical region, where it occurs in the Veracruzan Province but is restricted to the Sierra Norte de Chiapas subprovince. The type locality has an altitude of approximately 740 m, a tropical rainforest climate (Af) ( García and CONABIO 1998), and a tropical evergreen forest vegetation type.

## ﻿Conclusion

Previously, 15 extant (Bueno-Soria 2010) and one fossil (Wichard et al. 2006) species of *Xiphocentron* were known from Mexico. With the addition of three new species described here, the number of *Xiphocentron* species known from Mexico is now 19. However, many species are still only known from their type locality, and many regions remain poorly explored for the genus, particularly the Sierra Madre Occidental and Sierra Madre del Sur along the Pacific Coast. Therefore, more collections are necessary to obtain a better idea of the distribution and actual diversity of the genus *Xiphocentron* in Mexico.

## Supplementary Material

XML Treatment for Xiphocentron (Glyphocentron) flinti

XML Treatment for Xiphocentron (Antillotrichia) holzenthali

XML Treatment for Xiphocentron (Antillotrichia) pineroi

XML Treatment for Xiphocentron (Antillotrichia) rhamnes
